# Early identification of hERG liability in drug discovery programs by automated patch clamp

**DOI:** 10.3389/fphar.2014.00203

**Published:** 2014-09-02

**Authors:** Timm Danker, Clemens Möller

**Affiliations:** ^1^NMI TT GmbHReutlingen, Germany; ^2^Life Sciences Faculty, Albstadt-Sigmaringen University of Applied SciencesSigmaringen, Germany

**Keywords:** hERG, Torsades de Pointes, cardiac arrhythmia, electrophysiology, automated patch clamp, safety pharmacology, ion channel, ADMET

## Abstract

Blockade of the cardiac ion channel coded by human ether-à-gogo-related gene (hERG) can lead to cardiac arrhythmia, which has become a major concern in drug discovery and development. Automated electrophysiological patch clamp allows assessment of hERG channel effects early in drug development to aid medicinal chemistry programs and has become routine in pharmaceutical companies. However, a number of potential sources of errors in setting up hERG channel assays by automated patch clamp can lead to misinterpretation of data or false effects being reported. This article describes protocols for automated electrophysiology screening of compound effects on the hERG channel current. Protocol details and the translation of criteria known from manual patch clamp experiments to automated patch clamp experiments to achieve good quality data are emphasized. Typical pitfalls and artifacts that may lead to misinterpretation of data are discussed. While this article focuses on hERG channel recordings using the QPatch (Sophion A/S, Copenhagen, Denmark) technology, many of the assay and protocol details given in this article can be transferred for setting up different ion channel assays by automated patch clamp and are similar on other planar patch clamp platforms.

## INTRODUCTION

A number of drug discovery and development programs have been hampered by issues with drug induced cardiac arrhythmia. This is particularly well-known for histamine receptor antagonists, e.g., the potent human ether-à-gogo-related gene (hERG) channel blocker Terfenadine (Teldane^®^, Seldane^®^). Terfenadine is an H1 receptor antagonist that was launched in 1982 and was later withdrawn from the market because it potentially caused a life-threatening ventricular tachyarrhythmia, torsades de pointes. The potential to affect cardiac ion channel currents and thereby potentially induce cardiac arrhythmia can occur for compounds from many different chemical classes and in very different therapeutic areas.

### hERG CHANNEL BLOCKADE CAN CAUSE CARDIAC ARRHYTHMIA

Nearly 20 years ago it was found that mutations in the hERG could cause long QT syndrome. This inherited disorder can be observed in the electrocardiogram as prolonged QT interval and is correlated to torsades de pointes ventricular tachyarrhythmia (Curran et al., 1995; Sanguinetti et al., 1995; Sanguinetti and Tristani-Firouzi, 2006). The protein coded by hERG was later identified as a potassium-selective cardiac ion channel, Kv11.1, which plays an important role during cardiac repolarization. Consequently, malfunction of this ion channel may cause a delay in cardiac repolarization. The importance of the hERG ion channel for drug discovery programs stems from the observation that not only mutations, but also drug induced blockade of the channel, may cause repolarization abnormalities. Many drugs from different chemical classes and therapeutic areas have been shown to block the hERG-coded ion channel and may in turn potentially trigger torsades (Bruin et al., 2005). Other reasons for cardiac arrhythmias have been identified (Gintant and Hoffman, 1984; Roden et al., 1996; Fenichel et al., 2004; Gintant et al., 2011), but hERG channel blockade has become the most frequent single cause for drug withdrawals (Fenichel et al., 2004), and many drug discovery programs have been delayed (imposing significant costs on the pharmaceutical company) or stopped due to hERG channel liabilities of potential drug candidates.

Examples of compounds that show undesired hERG channel activity include Terfenadine, Astemizole (Zhou et al., 1999), brompheniramine (Park et al., 2008) and many other drugs as listed on, e.g., http://crediblemeds.org/everyone/composite-list-all-qtdrugs/, a website maintained by the independent non-profit organization AZCERT, Arizona, and currently sponsored by the Science Foundation Arizona. Reduction of hERG channel liabilities is continuously and extensively discussed in the literature (Ito, 2009; Anderson et al., 2010; Davenport et al., 2010; Levoin et al., 2011; Tye et al., 2011; Bahl et al., 2012; Becknell et al., 2012; Hudkins et al., 2012; Moorthy et al., 2014). While compounds from very different chemical classes may interact with the hERG channel due to its relatively large hydrophobic pore, the property of hERG channel liability has been observed especially often for histamine receptor antagonists. The reason for this is that the pharmacophores of the hERG channel and the histamine receptor show remarkable similarities (Davenport et al., 2010).

### AUTOMATED PATCH CLAMP ELECTROPHYSIOLOGY IS AN EMERGING TECHNIQUE FOR IDENTIFYING CARDIAC ION CHANNEL LIABILITIES

To identify potential hERG liabilities early in drug discovery programs and thus avoid problems with hERG channel interactions for late-stage compounds, it has become common practice in drug discovery programs to start testing compounds relatively early during the drug discovery process on potential hERG channel blockade. Finally, before administration to humans, the ICH S7B guideline requests compounds to be tested on potential repolarization issues under GLP, typically employing electrophysiological patch clamp assays which are considered the “gold-standard” for ion channel investigations.

A number of assays have been developed to gain a picture of compound effects on the cardiac action potential and in particular on repolarization effects. These assays include hERG ion channel assays employing (but not being limited to) fluorescence, binding, atomic absorption or electrophysiological techniques measuring interaction with, or the function of, the hERG ion channel, with the different throughput inherent to these techniques. In addition, assays have been developed that assess the effects of compounds on other cardiac ion channels. These data may add important information and draw a more complete picture on cardiac effects than possible with hERG channel data alone, since effects on other cardiac ion channels may potentiate or reduce hERG channel effects.

Also, assays that could potentially deliver more physiologically relevant data than assays relying on only one ion channel type are used. In the past years, techniques based on stem cell derived cardiomyocytes particularly gained significant interest (Hamdam et al., 2013). Such assays integrate effects on several ion channels beyond the hERG channel. Therefore effects on several channels and interactions of such effects might be detected in assays using these preparations.

Despite these efforts in developing, validating, and employing more complex assays (which might be considered especially important for valuable late-stage compounds), the hERG ion channel remains a major potential trouble maker in drug discovery programs. It is therefore not surprising that interest in this ion channel was one of the major drivers in developing automated electrophysiology patch clamp instrumentation (Dunlop et al., 2008). Automated patch clamp assays have been successfully used in a number of drug discovery programs, both for identifying compound effects on ion channel targets as well as for identifying undesired off-target effects. Technical advances in the automated patch clamp technique and in cell preparations have facilitated employing automated patch clamp assays with increasing success rates (Finkel et al., 2006; Zeng et al., 2008), high parallelization (and, consequently, increased throughput), at (or near) physiological temperature (Polonchuk, 2012), at increased ligand application rates, and also using more challenging cell preparations such as primary cells on these instruments (Farre et al., 2009; Jones et al., 2009; Milligan et al., 2009; Golden et al., 2011; Stoelzle et al., 2011a,b; Haythornthwaite et al., 2012; Becker et al., 2013; Gillie et al., 2013; Milligan and Möller, 2013). The development of higher throughput automated patch clamp instruments has allowed moving electrophysiological ion channel assays earlier into the drug discovery process and has made electrophysiology measurements compatible with medicinal chemistry iteration cycles. This is especially important for those drug discovery programs in which hERG channel interactions could be expected from earlier experiences with certain target (such as histamine receptor programs, as noted above) or compound classes.

In this manuscript we discuss protocols particularly suited for measuring hERG ion channel effects during the Hit-to-Lead and Lead Optimization phases in drug discovery programs. While during high-throughput screening a small percentage of false negatives or false positives could potentially be tolerated, this is generally less acceptable during later stages of compound development, as false or misinterpreted data might guide medicinal chemistry programs in the wrong direction. Therefore, for this article, we focus on using medium throughput automated patch clamp instrumentation, such as the QPatch^TM^ (Sophion Biosciences A/S, Copenhagen) or the Patchliner (Nanion Technologies GmbH, Munich, Germany) automated electrophysiology platforms, to deliver high data quality. These instruments allow 2 to 8 (Patchliner) or 8 to 48 (QPatch) parallel high quality measurements of individual cells in the gigaseal configuration. Importantly, many of the notes and caveats discussed in this manuscript stem from general (and partially basic) electrophysiological considerations. Therefore, this basic electrophysiology, as well as cell preparation details, applies to most of the other automated patch clamp platforms [e.g., SyncroPatch 96 and SyncroPatch 384 PE (Nanion Technologies GmbH, Munich); PatchXpress, IonWorks Quattro, Barracuda and Barracuda Plus (MDS, Sunnyvale, CA, USA); IonFlux HT (Fluxion Bioscience Inc, San Francisco, CA, USA), Qube (Sophion Biosciences A/S, Copenhagen)], and required validation steps are very similar between the different instruments. Other protocol details for measuring more challenging cell types, in particular for cardiac safety evaluations using stem cell derived cardiomyocytes using the Patchliner, may be found in other articles (Stoelzle et al., 2011a; Milligan and Möller, 2013). Protocol optimization for other ion channels can be guided along some of the notes discussed in this article.

## MATERIALS

### CELL LINE

A commercially available cell line (“CHO hERG DUO,” BSys, Switzerland) was used (note 1).

### CELL CULTURE AND CELL HARVESTING REAGENTS

#### Cell culture medium

450 ml HAM’s F-12 + Glutamax (Invitrogen, 31765)

+50 ml FBS Gold (PAA, A15-151)

+1 ml G418 = 100 μg/ml (PAA, P11-012)

+1 ml Hygromycin 100 μg/ml (PAA, P02-015)

#### Serum free medium

25 ml CHO-S-SFM I (Invitrogen, 12052)

+25 mM HEPES

+0.04 mg/ml Soy bean trypsin inhibtor (Sigma, T65222)

+100 unit/ml Penicillin/Streptomycin [(P/S) Invitrogen15140]

#### Thawing medium

450 ml HAM’s F-12 + Glutamax (Invitrogen, 31765)

+50 ml FBS Gold (PAA, A15-151)

#### Harvesting agent

Accutase [PAA (L11-007)]

#### Recording Buffers

Extracellular recording buffer (EC), in mM: 145 NaCl, 4 KCl, 1MgCl2, 2 CaCl2, 10 HEPES, 10 Glucose; adjust pH to 7.4 with NaOH.

Intracellular recording buffer (IC), in mM: 120 KCl, 10 HEPES, 5 CaCl2, 1.7 MgCl2, 4 K2ATP, 10 EGTA; adjust pH to 7.2 with KOH, osmolarity to 292 mOsm with Saccharose.

## METHODS

### CELL CULTURE PROTOCOLS

#### Culturing of cells from frozen vials

(1) Prepare 35 ml cell culture thawing medium.
(2) Thaw the vial quickly in a 37°C water bath and add to thawing medium.
(3) Transfer the thawing medium with the cells to a T175 flask containing pre-heated culture medium.
(4) After 3–4 h, exchange thawing medium with cell culture medium.
(5) Sub-culture after 2 days.

#### Sub-culturing

(1) Remove culture medium.
(2) Wash with 10 ml PBS.
(3) Remove PBS and add 2 ml Accutase.
(4) Incubate at room temperature (∼4 min).
(5) Make sure that the cells have a round shape before tapping.
(6) Gently tap on the side of the flask and add 5–7 ml culture medium and resuspend the cells by working the cell suspension up and down 5–10 times.
(7) Determine the cell density and viability by counting the cells in a Hemocytometer using Trypan Blue.
(8) Add the number of cells to the mother flask and the experiment flasks according to the subculturing plan below.
(9) Grow the cells at 37°C, 5% CO_2_ until roughly 80% of the available surface is covered with cells, corresponding to a confluency of the cells of 80%.

#### Sub-culturing plan for making mother flasks and experiment flasks

(1) Add 1.85 × 10^6^ cells per T175 flask for sub-culturing/experiments after 48 h.
(2) Add 0.8 × 10^6^ cells per T175 flask for sub-culturing/experiments after 72 h.
(3) Add 0.3 × 10^6^ cells per T175 flask for sub-culturing/experiments after 96 h.

#### Cell harvesting for automated electrophysiology patch clamp experiments (for T175 flask)

The cell harvesting for the automated patch clamp experiment should be carried out right before the start of the experiment. Typically, good results can be obtained until up to 4 h after preparation.

The aim of the cell harvesting procedure is to bring the adherently growing cells into suspension while fully maintaining their viability and physiological properties. After preparation, the cell suspension should consist of isolated, single cells. The abundant presence of cell clusters as well as fragments from dead cells would inevitably lead to unsatisfying success rates.

(1) Remove culture medium and wash with 7 ml PBS.
(2) Add 2 ml Accutase per T175 culture flask.
(3) Incubate the culture flask at room temperature for ∼4 min (ensure that the cells have reached a round shape and begin to detach before proceeding to the next step; see note 2).
(4) Gently tap on the side of the culture flask a few times. This should at this time be sufficient to detach almost all of the cells from the flask bottom.
(5) Add 8 ml serum free medium and resuspend the cells by slowly working the cell suspension up and down 5–10 times. This step is crucial to allow a precise counting in the next step. Resuspend just as vigorous as needed to obtain single cells and to avoid cell clusters. Don’t do more than needed since this might damage some of the cells. Avoid air bubbles.
(6) Determine the cell density and viability by diluting an aliquot 1:2 in Trypan Blue and count the cells in a Hemocytometer. Also determine the number of cell clusters vs. number of single cells.
(7) Adjust cell density to 3 million cells per ml. You should gain at least 12 ml of cell suspension which are then placed in the cell container of the QPatch (“QStirrer”).

### PREPARATION OF TEST COMPOUND SAMPLE DILUTIONS

For testing with the automated patch clamp systems, dilutions of the test samples in the final test concentration have to be prepared and placed in a 96-well plate. This so called “compound plate” should be prepared immediately before the experiment. The plate can be either a plastic disposable or a reusable tray, accommodating glass inserts. The latter is recommended, since certain types of test compounds tend to adhere to the walls of plastic ware, which may reduce the concentration actually being tested (see also note 7).

### AUTOMATED ELECTROPHYSIOLOGICAL RECORDINGS

After loading the compound plates and the harvested cells into the instrument, a previously defined assay protocol is started to carry out the measurements. The assay protocol contains cell type specific settings for establishing the patch clamp recording configuration, as well as “voltage protocols” to elicit the ion channel currents and “application protocols” that determine the order and timing of drug applications.

While the settings to achieve the recording configuration for most common hERG expressing cell lines are typically provided by the instrument supplier, and are therefore not discussed here, we will provide detailed information about setting up the voltage and application protocols in the following sections.

#### Voltage stimulation protocol for hERG

As a voltage gated channel, the hERG channel can be opened and closed by varying the membrane potential. This is accomplished by applying appropriate voltage stimulation protocols, which has to be set up in the appropriate software section of the instrument.

**FIGURE 1:**
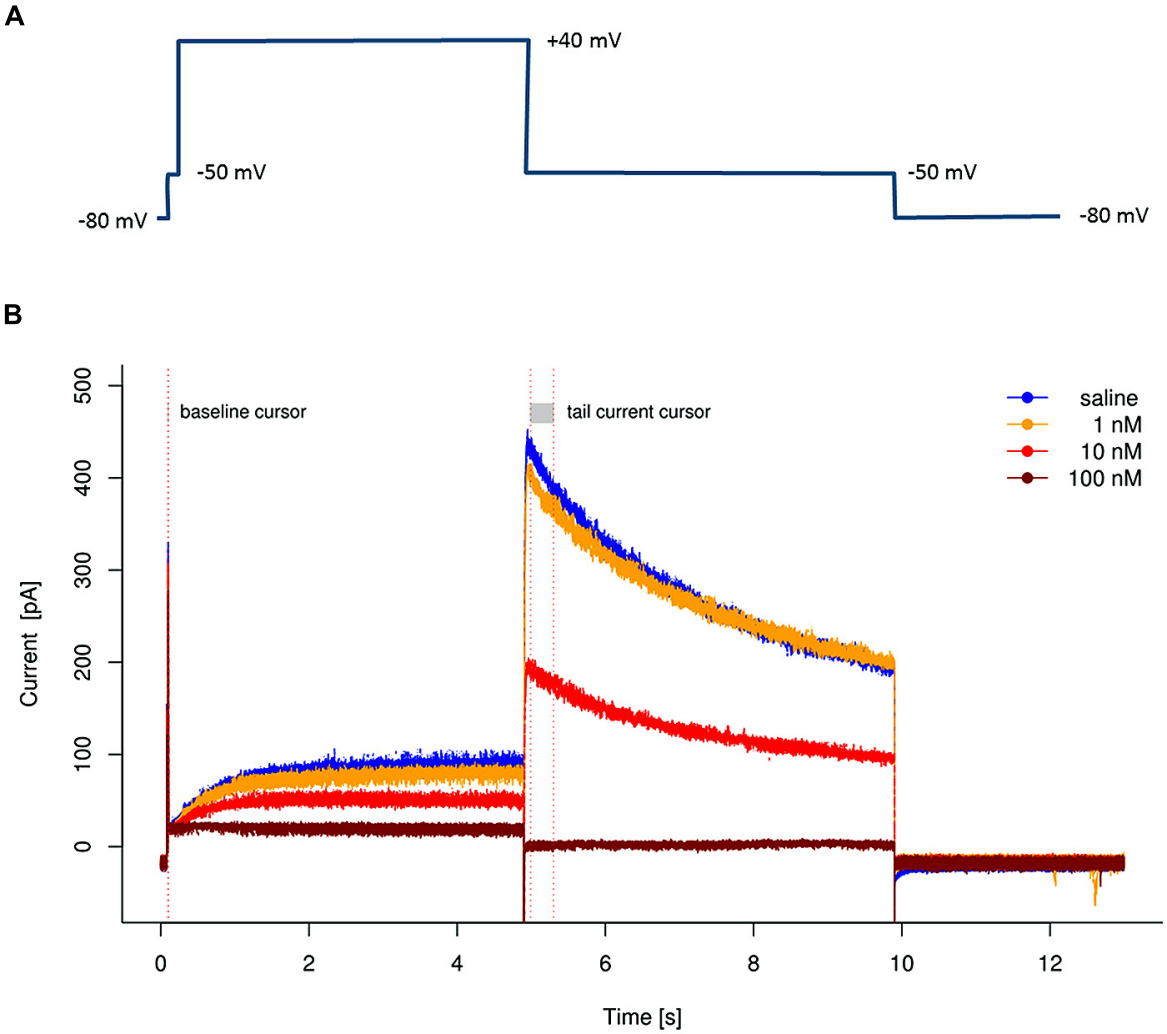
**(A)** Stimulation protocol for hERG channel measurements. **(B)** Example of hERG recordings showing the effect of Cisapride. Four representative traces from the same cell are superimposed to visualize the effect of increasing concentrations of the known hERG blocker Cisapride.

Typical for voltage gated potassium channels, the hERG channels opens and partially inactivates at positive voltages. However, in contrast to most other potassium voltage gated channels, the hERG channel shows a very prominent tail current at repolarization which can be even larger than the current recorded during depolarization. The protocol shown in **Figure 1A** shows a widely used stimulation protocol.

From a holding potential of -80 mV, the cell is briefly clamped at -50 mV to test leak current at this potential with closed hERG channels (“baseline step”). This is followed by a depolarization to +40 mV, where the hERG channels are alternating between the open and the inactivated state. After returning to -50 mV, the inactivated subpopulation of hERG channels rapidly recovers from inactivation and switches into the open state, from where the channels slowly close. This results in a large tail current to be observed in the second -50 mV phase.

A cell expressing hERG channels will respond to this stimulation protocol with a typical current signature, which can be unequivocally identified as a hERG current, as shown in **Figure 1B**.

To quantify the hERG current amplitude, values for “baseline” and “peak tail current” are determined by averaging the recorded currents during the baseline step and at the beginning of the second – 50 mV period [these time intervals are marked by the red dotted lines (“cursors”) in **Figure 1B**]. The hERG channels are in different states during the two -50 mV phases. In the phase preceding the baseline measurement, the cell is clamped at holding potential and the hERG channels are fully closed. The potential of -50 mV is not sufficient to open closed hERG channels, so the entire channel population of the cell remains closed. Any current recorded during this period is therefore considered not originating from hERG channels (“baseline current” or “leak current”). In contrast, after the depolarization to +40 mV, some of the hERG channels are in an open state, but most of them are in a non-conducting, inactivated state. After repolarizing back to -50 mV, recovery from inactivation is a very fast process. Therefore, the hERG current reaches its highest peak at the beginning of the repolarization to -50 mV (“peak tail current”). The “baseline” level measured before, representing the isolated leak current, will be subtracted from the peak tail current for correction.

#### Application protocols

**FIGURE 2:**
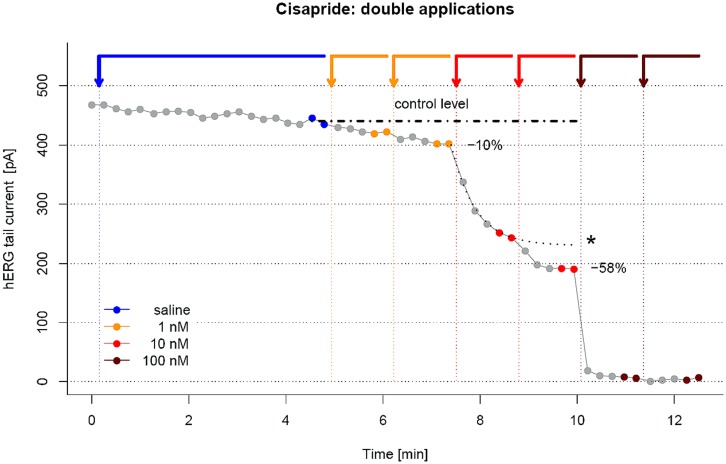
**Current vs. time (I/t) plot of a three point dose-response assessment of Cisapride on hERG currents.** Downward arrows at the top of the figure mark time points where saline or test compound is applied, and horizontal bars indicate the duration of exposure. The last two data points before the next application (marked by colored points) are averaged to calculate the effect of the preceding drug application. Drug applications are performed twice to ensure saturation of drug effect, where only the second drug application is used for data analysis.

For evaluation of drug effects, after establishment of the recording configuration the voltage stimulation protocols are performed in equidistant time intervals of 15 s while different liquid solutions are applied to the cell. For analysis, the current amplitudes of each recorded stimulation protocol can be plotted vs. time. **Figure 2** shows an example of a standard application protocol. After a stabilization period, fresh saline is applied to the cell and a control recording period of at least 3 min should be recorded. Current amplitude at beginning and end of this control period should show no significant change. The current amplitude at the end of the control period is then used as a reference point for the measured currents after drug application. (It is good practice to apply each drug concentration multiple times, e.g., twice, as shown in **Figure 2**; see notes 3, 6, and 7.)

#### Data filtering and dose-response analysis

A typical dose-response curve (DRC) for hERG channel testing will be calculated from a minimum of three and up to eight different drug concentrations, where each drug concentration will be tested on at least three different cells. While it is common practice to test more than one drug concentration on a single cell (as shown in **Figure 2**), the number of concentrations applied to a cell is limited by the life time of the electrophysiological recording, which is often not more than 30–40 min. After this period, relevant quality criteria of the recording (see notes 4, 5) tend to degrade. On the other hand, each drug application should be given enough time to allow the drug effect to reach steady state (see note 6). Therefore, the number of test concentrations that are applied to a cell is limited. The combination of the number of drug concentrations to be tested and a sufficiently long drug exposure time must not exceed the average life time for a stable, high quality recording in whole cell configuration.

**FIGURE 3:**
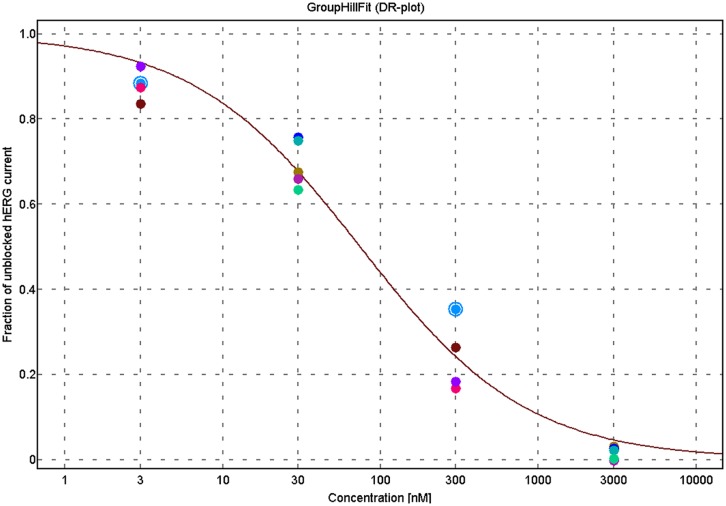
**Dose-response fit through the data of nine cells (each identified by a unique color) with two concentrations of Terfenadine applied to each cell.** For each cell, hERG tail current values representative for each compound concentration where determined as shown in **Figure 3**, and normalized to the unblocked hERG current value which was recorded at the end of the saline period before the first compound application. The combined data of all nine cells was then fitted to the hill equation y = c^h^/(IC_50_^h^+c^h^) to estimate the half maximal inhibition concentration (IC_50_ value) and hill coefficient h for this compound (IC_50_ = 72 nM, *h* = 0.8).

The test concentrations can be distributed over several cells and the data recombined during analysis. The built in data analysis of the QPatch software is capable to automatically group all data from cells that where treated with the same compound and to calculate the DRCs accordingly (see **Figure 3**). However, a thorough quality control which filters out all low quality recordings should precede this step.

A recording from a cell that qualifies for inclusion into further data analysis, e.g., for DRC fitting (**Figure 3**) should meet at least the following criteria (see also note 9):

(1) The membrane resistance (Rm) should not fall below a given threshold (e.g., 500 MΩ) at any time (see note 4)
(2) The series resistance (Rs) should not exceed a given threshold (e.g., 10 MΩ) at any time (see note 5)
(3) The initial I_hERG peak tail current should not be smaller than a given value (e.g., 250 pA)
(4) The leak current should not be greater than 20% of the inital hERG current at any time
(5) The change in current during the control phase (“run down” or “run up”), should not exceed a given value (e.g., ±10%), see note 8.

To demonstrate the effect of quality control using the key parameters Rm and Rs, we analyzed the percentage of remaining hERG current after treatment with 100 nM Terfenadine in a test data set of 22 recordings (see **Table 1**). Quality control parameters for Rm and Rs where defined and used to divide the test set into an “accepted” and a “rejected” subgroup, and the mean and standard deviation (SD) of relative remaining currents for each subgroup was calculated. **Table 1** shows the effects of applying quality criteria for Rm, Rs, or both. The subsets with good Rm and Rs values show reduced SD and more reliable data. Therefore, especially during routine screens with small sample sizes, excluding data with poor Rs or Rm is highly recommended.

**Table 1:** Influence of quality control on the relative remaining hERG current after block with 100 nM Terfenadine.

Quality criterium	Relative remaining current after application of 100 nM Terfenadine	
	Accepted	Rejected
Rm > 150 MΩ	60 ± 13% (*n* = 18)	80 ± 78% (*n* = 4)
Rs < 15 MΩ	61 ± 15% (*n* = 16)	70 ± 60% (*n* = 6)
Rm > 150 MΩ and Rs < 15 MΩ	57 ± 11% (*n* = 14)	74 ± 52% (*n* = 8)

*A test set of 22 cells was treated with 100 nM Terfenadine and the relative remaining hERG current was measured. Different quality criteria were applied to divide the test set into two subgroups, named “accepted” and “rejected.” For each criterium, the results of the subgroups are given as mean ± SD (number of cells).*

On the QPatch and Patchliner assay softwares, these quality criteria can be conveniently checked by setting up a customized plot which shows I_hERG, Rm, Rs on a combined panel. Alternatively, these can be set up as automated quality controls, based on user defined so called “data filters.” Such data filters enable the user to deal with voluminous amounts of data typical for large projects.

Of course, rejecting a large number of cells comes at a significant cost. In large screens, a statistical analysis of test datasets may help to find the exact thresholds for quality criteria in order to balance data quality against economics.

## NOTES

### NOTE 1: CELL LINE

A number of excellent validated hERG channel expressing cell lines are commercially available (e.g., from ChanTest, Cleveland, OH, USA; Millipore Merck KGaA, Darmstadt, Germany; CCS Cell Culture Service, Hamburg, Germany; bSys, Basel, Switzerland; Anaxon, Berne, Switzerland), or can be constructed using well-established molecular biology techniques. Also, cell lines conveniently provided as frozen cells (Donovan et al., 2011) are available (e.g., from Cytocentrics, Rostock, Germany and CCS Cell Culture Service, Hamburg, Germany). Especially with the possibility to patch cells directly from frozen stocks, these greatly ease the requirements for cell culture works making cell culture a much more manageable process. When deciding on a cell line, the channel expression rate in suspension should be considered, as this may be significantly different from the expression rate in adherent cells. When channel expression is low after cell harvesting, FCS in the medium as well as incubating and growing the cells at lower temperature can help to recover the current. With some cell lines we have made good experience with incubating the cells at 8°C for 1–3 h after harvesting before the patch clamp experiment, which has increased the hERG current amplitudes. Vendors of automated electrophysiology instruments make recommendations on cell lines suitable for their instruments, and cell line vendors provide validation data of their lines used on planar patch clamp systems.

It has been discussed whether cell lines based on CHO cells or HEK cells provide more relevant data (Wiśniowska and Polak, 2009). Independent of which cell type is used, however, it is vital to validate the channel response well, including data of reference compounds, and to make sure that no non-hERG background currents confound the data.

### NOTE 2: CELL DETACHMENT

Quality of the cells will critically depend on incubation time in the cell detachment agent before harvesting. When the incubation is too short, cells will not detach from the flask easily and detachment will require a great extent of mechanical interaction, like knocking on the side of the flask. As a result, cells will be damaged when they detach from the flask, resulting in poor success rates in the patch clamp experiments. The amount of cell damage can also easily assessed by a viability staining of the cell preparation with trypan blue and should be <5%. When cells are incubated for too long, the seal rate in experiments is often observed to be excellent; however, seal stability often becomes poor.

### NOTE 3: USE OF DOUBLE DRUG APPLICATIONS

In the example given in **Figure 2**, each drug concentration is applied twice (“double drug application”). From the example given, it is easy to see that extrapolation of the time course of compound effect following the first drug application (dashed lines and asterisk) shows a significant difference to the actual time course after the second drug application of the same concentration, demonstrating that double drug applications can give more accurate results compared to single drug applications. This is particularly important for hydrophobic (“sticky”) compounds, where even more (sometimes more than four) compound applications may be required before steady state of compound effect is reached, see note 7 and Figures **Figure 2** and **Figure 6**.

### NOTE 4: PROBLEMS INTRODUCED BY INSUFFICIENT MEMBRANE RESISTANCE

The total membrane resistance (Rm) of an intact, healthy cell with closed ion channels is in the order of several GΩ. When the cell is placed on a patch clamp chip and recording configuration is established, a fraction of the cells membrane is sucked into a small hole in the surface of the chip. While the cell membrane in contact with the rim of the hole ideally forms an electrically tight seal with the material of the chip, the membrane in the center of the hole finally becomes ruptured, which allows contact of the amplifier to the interior of the cell through the hole. In this recording configuration (the so called “whole cell” configuration), a high value of the measured membrane resistance indicates that the rest of the cell membrane as well as the cells contact to the chip are intact. This is the prerequisite of high quality recordings. Under conditions where the hERG channels are closed, for example at holding potential or during the baseline step, almost no “leak” currents should be visible in the recording, indicating a high Rm.

**FIGURE 4:**
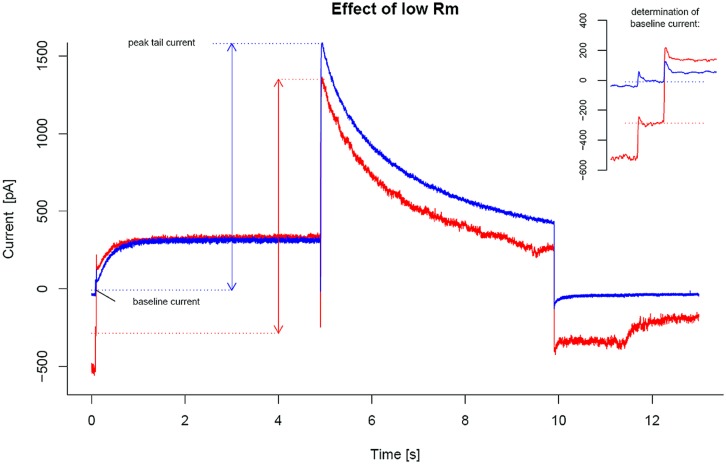
**Effect of small membrane resistance (Rm).** Two superimposed hERG recordings from a cell with instable membrane resistance are shown. The blue trace represents an early recording from this cell, where Rm has a high value in the GΩ range. The red trace was recorded from the same cell after Rm dropped to ∼100 MΩ. The baseline (or “leak”) currents recorded during the brief -50 mV “baseline step” are indicated by horizontal dotted lines. The inset in the upper right corner provides a zoomed view on how the baseline current is determined during the baseline step. Due to the drop in Rm, baseline current and peak tail current are shifted to a comparable extent. The difference between baseline and peak tail current, indicated by the double headed vertical arrows, is more robust to changes in Rm than uncorrected peak tail current alone.

In **Figure 4**, two example recordings from the same cell, with different Rm, are compared. The blue trace is recorded shortly after obtaining the whole cell configuration. It represents a cell with a good membrane resistance in the GΩ range. At holding potential (begin and end of the trace) and during the baseline step the recorded “leak” current is close to zero. In contrast, the red trace shows a hERG recording from the same cell after Rm had dropped to ∼100 MΩ. The uncorrected peak tail current has dropped from 1600 to 1300 pA. At holding potential and during the baseline step, the “leak” currents deviate significantly from zero. The peak tail current can be corrected with the baseline level (as recommended), the corrected peak tail current will be less affected. However, with increasing degradation of Rm the results will finally become highly inaccurate.

### NOTE 5: PROBLEMS INTRODUCED BY LARGE SERIES RESISTANCE

The series resistance (Rs), also commonly referred to as access resistance, is the electrical resistance between the amplifier input and the cell membrane of the recorded cell. In automated patch clamp, it is largely determined by the opening diameter of the hole in the patch clamp chip that makes contact to the cell. However, Rs may further be increased when the membrane that spans the hole is only partially ruptured, or for example when cell organelles are being drawn into the hole. With Rs being very large, the amplifier is not able to fully control the electrical potential of the cell membrane. As a result, the actual cell membrane potential may deviate significantly from the potentials defined by the voltage stimulation protocol. The ion channels will therefore not be accurately stimulated, and thus their current responses will be altered.

**FIGURE 5:**
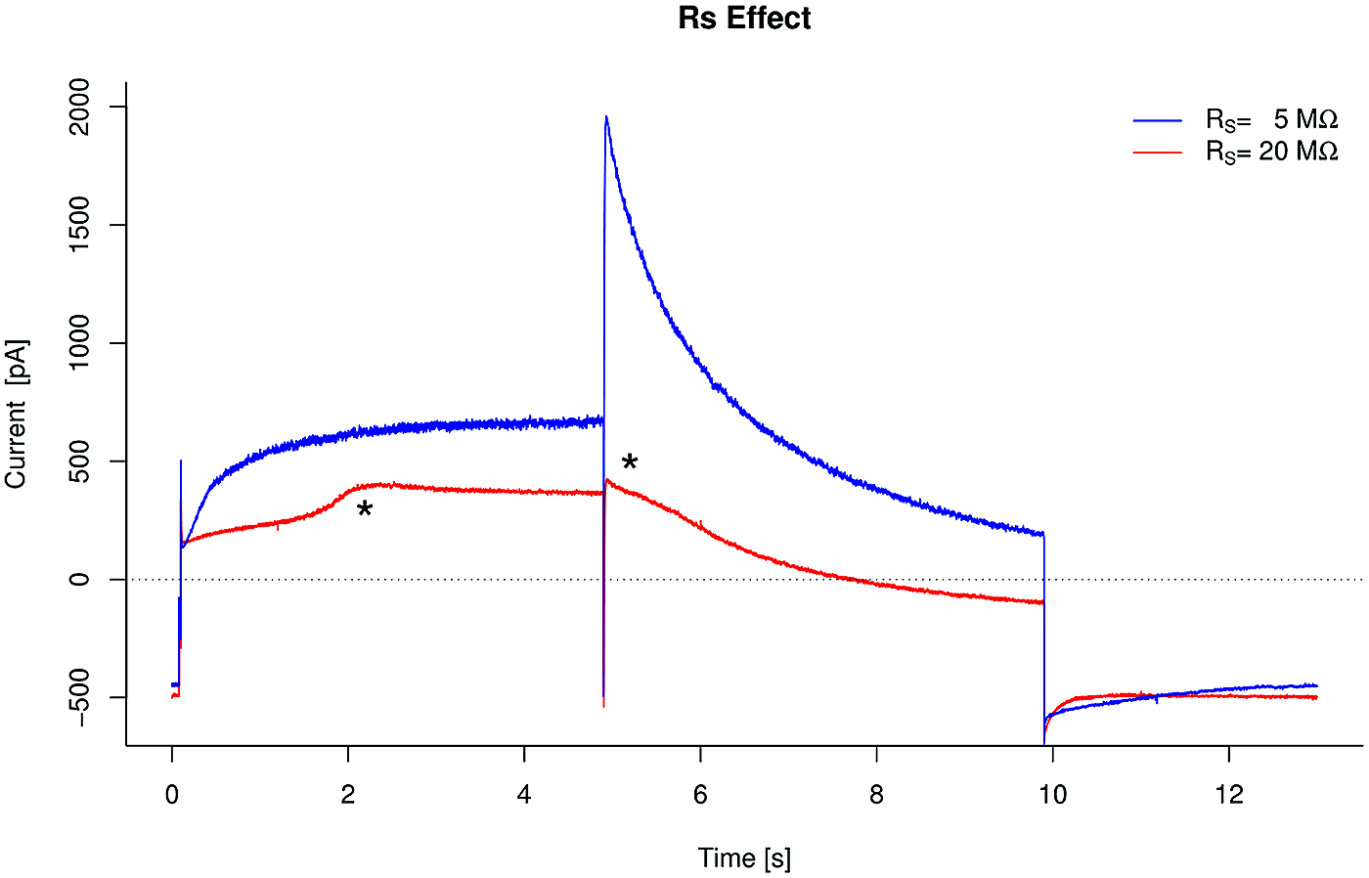
**Effect of series resistance increase on hERG measurements.** In this drastic example, a change in series resistance (Rs), combined with a stable, but critically low membrane resistance (Rm = 120 MΩ), leads to a significant distortion in the readout.

**Figure 5** shows an example of the typical artifacts introduced by large Rs when recording hERG currents.

As a rule of thumb, when doing dose-response recordings, Rs should be as small as possible (preferably < 10 MΩ) and stable over time.

### NOTE 6: INCOMPLETE STEADY STATE OF DRUG EFFECTS

The time needed for a compound effect to reach steady state varies from compound to compound.

Failure to achieve steady state before applying the next compound concentration will lead to underestimation of the drug effect and therefore to artificially right-shifted DRCs.

Whether a sufficient steady state has been reached can be judged by examining the I/t plot of the recording.

Terfenadine is an example of a compound for which the results of IC_50_ measurements critically depend on experimental conditions. For manual patch clamp measurements, 23-fold differences have been found in literature values (Kirsch et al., 2004). Due to its hydrophobic or “sticky” nature, the compound is particularly difficult to measure accurately with automated patch clamp devices (see note 7). Among several known hERG inhibitors tested (Guo and Guthrie, 2005), Terfenadine exhibited one of the largest discrepancies (10 vs. 77 nM) in hERG IC_50_ values when comparing standard patch clamp data to automated data. The compound also reaches steady state of drug block very slowly, so that prolonged drug exposure times have to be considered for accurate dose-response assessments.

**FIGURE 6:**
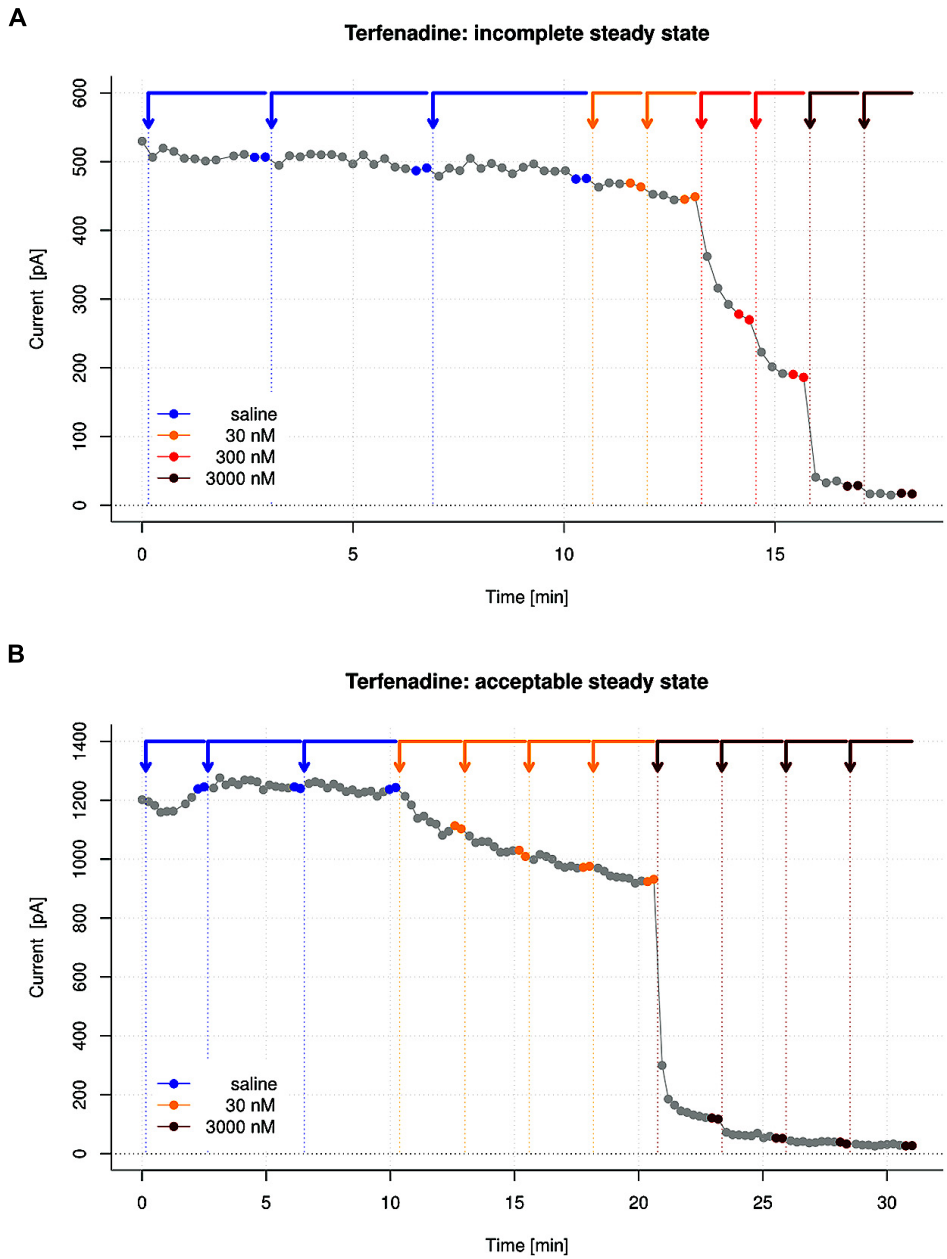
**Good and bad practice examples for steady state of drug induced block by Terfenadine. (A)** Bad practice example of an I/t plot showing insufficient steady state of block, which would lead to underestimation of the drug effect. **(B)** Good practice example where the drug exposure time as well as the number of drug application repeats has been doubled, leading to an acceptable steady state.

In the “bad practice” example shown in **Figure 6A**, the blocking effect of Terfenadine is evaluated with the same protocol as in **Figure 2**. With the first applied drug concentration of 30 nM, almost no block is reported by the assay. Analysis of the I/t plot clearly reveals that the drug effect develops very slowly, so that steady state is not reached before the next drug concentration is applied. Increasing the drug application time increases current blockade, and repeated drug applications improve the actual drug concentration at the target. A “good practice” example is shown in **Figure 6B**, where the number of repeated drug applications and the drug exposure time is doubled, an acceptable steady state is reached, and a significant drug induced block of 30% is found for the same concentration.

Therefore, the assay scheme in the first example leads to an underestimation of the compound effect. A prolongation of the time periods between the drug applications leads to more accurate IC_50_ determinations for such slowly acting compounds.

To demonstrate the effect of incomplete steady state, we analyzed the effect of a prolonged drug exposure to Terfenadine.

The recording shown in **Figure 6A** is representative for a test data set of seven recordings featuring a drug exposure time of 150 s per compound. The average IC_50_ for Terfenadine in this data set is 180 nM (*n* = 7). In contrast, when we doubled the drug exposure time, as shown in **Figure 6B**, we obtained an average IC_50_ of 72 nM under otherwise similar conditions (see **Figure 3**).

### NOTE 7: RECOMMENDATIONS FOR STICKY COMPOUNDS

Some compound classes are difficult to dissolve in aqueous solutions and also known for their notorious “sticky” behavior, typically a result of their hydrophobic nature. It has been commonly observed that with these compounds, the actual applied concentration is in fact reduced by adherence of compound molecules to the walls of the containers where the compound dilutions are stored in. The result of such effects would be an artificially right-shifted DRC, and therefore may lead to failure of detection of problematic hERG effects.

To reduce such unwanted effects, the following precautions are recommended:

• Use glassware as a standard for compound plates, vials, and even pipette tips.
• Set up the dilutions in rather large vials (at least 1 ml).
• Prepare a DMSO stock solution at a 1000 fold higher concentration than the maximum tested concentration.
• To prepare the various test concentrations, dilute the stock solution first with DMSO, then dilute 1:1000 with EC, thus keeping the final DMSO concentration at a constant level of 0.1%. An increased DMSO concentration of up to 1% may be tolerated in your assay.
• If feasible prepare compound dilutions manually and closely look for precipitation.
• Prepare the compound plate immediately before running the experiment.

It may be required to analyze solubility and final concentrations of compounds in buffer solutions using, e.g., LC/MS (when available).

### NOTE 8: RUN DOWN

From time to time, a downward trend of the hERG current amplitudes during the control phase, i.e., independent of any drug application, may be observed. This phenomenon is commonly referred to as “run down”. Including cells exhibiting run down into dose-response analysis should be strictly avoided. Otherwise, it will result in artificially left-shifted DRCs, and therefore may lead to false positive identification of hERG blockade.

The main focus to fix run down problems should be the cell culture. Cell density in the culture flasks should not exceed 80% confluency, and CO_2_ concentration be kept sufficiently constant. Using an incubator that is frequently opened (i.e., because it accommodates cell cultures from different projects) may lead to CO_2_ fluctuations that might negatively affect cell quality.

It is also a good advice to check pH and osmolarity of IC and EC recording buffers before use, especially if frozen stocks are employed.

### NOTE 9: HILL COEFFICIENT

From dose-response (or IC_50_)-fits with a sufficient number of concentrations, the Hill coefficient can also be calculated with standard assay software packages. For many channel-compound interactions, the Hill coefficient would be expected to be close to 1. While other Hill coefficients are possible, these should be critically reviewed. Typical problems in experiments that can lead to Hill coefficients incorrectly deviating from one, and, consequently would produce incorrect IC_50_ values, are:

A Hill coefficient greater than one (steep IC_50_ curve) is often caused by underestimation of compound effects in particular at small concentrations, e.g., due to no steady state in compound effects (see note 6), absorption of compound to, e.g., tube materials (see note 7), and other reasons.

A Hill coefficient smaller than one often stems from underestimation of compound effects at high concentrations (e.g., due to poor voltage clamp conditions at large currents or large series resistance, see note 5), or overestimation of compound effects at low concentrations, e.g., due to run down effects (see note 8), that might be falsely interpreted as slow compound effects.

## OUTLOOK

This article focuses on protocols to identify cardiac safety issues caused by hERG ion channel blockade early in drug discovery programs by high quality medium throughput automated electrophysiology screening using cells expressing the hERG ion channel. The physiological significance of these recordings can be improved, at reduced throughput, by performing recordings at or near physiological temperature (Polonchuk, 2012). In addition to screening on the particularly important hERG ion channel, assessing the effects of compounds on other cardiac ion channels may be required. Recently, automated patch clamp screening of compound effects on the action potential in stem cell derived cardiomyocytes has become possible (Stoelzle et al., 2011a; Milligan and Möller, 2013). These assays provide information to further assess the cardiac safety of compounds. However, currently more validation of stem cell based assays is required before these can be routinely used for cardiac safety investigations.

Advances in the parallelization of patch clamp electrophysiology robots will further increase the throughput of patch clamp screening. A robot allowing parallel measurements of up to 768 wells with gigaseal and corresponding low leak currents is now available (Syncropatch PE, Nanion Technologies, Munich), and the throughput of patch clamp electrophysiology screening can be expected to increase even further.

## Conflict of Interest Statement

Timm Danker is employee of NMI TT GmbH, a company providing ion channel services.
